# Describing Racist or Racialised Actions Using the Declarative Mapping Sentence Method

**DOI:** 10.3389/fsoc.2022.779452

**Published:** 2022-04-14

**Authors:** Paul M. W. Hackett, Ava Gordley-Smith

**Affiliations:** ^1^Department of Philosophy, Nnamdi Azikiwe University, Awka, Nigeria; ^2^University of Wales Trinity Saint David, Lampeter, United Kingdom; ^3^School of Communication, Emerson College, Boston, MA, United States

**Keywords:** race, racism, racialization, declarative mapping sentence, Facet Theory, mapping sentence, definition

## Introduction

In the US and many other countries, when we watch or read the news, reports seem to demonstrate that there is an endless stream of racially motivated hateful behaviors and expressions. These are so commonplace that they may not make front page news. The physical attack upon, and murder of George Floyd in Minneapolis and the verbal attack on Christian Cooper in Central Park, New York City, both in May 2020, were exceptions that made the news headlines around the world. The prosecution of Floyd's killer, a serving police officer, demonstrated the increased prominence of racialized events that has resulted in raised awareness of racism in many communities. The above two incidents also show that racially motivated behaviors may be of very different kinds and forms.

Racism may also constitute a set of beliefs that embodies the notion that the social construct of race exists and that people from different races have divergent and separately identifiable qualities, characteristics, skills and abilities. Racism can take the form of prejudice, discrimination, or antagonism as this is expressed by individual's, institutions or even communities and nations. Racism may be targeted toward an individual person or a group of people due to their membership of a specific racial or ethnic group that usually possesses a marginalized or minority position in a society. Furthermore, the characteristics that a marginalized race is believed to possess are often used to identify such individuals as being inferior in relation to members of the race who are attributing such depreciatory characteristics.

Racism and racist acts are deeply repugnant and are disparaged by much of society. However, some of these behaviors and beliefs may be thought of as racialized rather than being expressly racist in that they may not be designed or intended to be negative toward a specific group, but even without this intention this is the consequence. Very often, the White establishment has largely ignored many of these institutional and social situations whilst concentrating upon the necessary and essential first-step of prohibiting racially abusive language and overtly racist acts.

An example of this can be seen in the effects of Covid-19 within the US which has impacted upon minority communities much harder than white communities. Being a person of color in the US has always been associated with a health status that is typically worse than that of white people and people of color have poorer health as measured along a series of measures (e.g., Ford, 2019). Gould and Wilson (2020) note how during the pandemic there has been a high level of association between being black and high incidences of Covid-19: “Black workers face the two most lethal preexisting conditions for coronavirus—racism and economic inequality. Black Americans make up 12.5% of the US population but account for 22.4% of COVID-19 deaths” (Gould and Wilson, 2020).

Being black has many other consequences. In terms of employment, many of the jobs that white people do not want to do are occupied by people of color and they are often paid less than the white people who do the same type of work. In the US, black individuals and other non-white people have a vastly higher probability of being stopped when driving by the police than do white drivers. There is also a very large and disproportionate number of people of color in US prisons and even more disturbingly, African Americans are nearly three times more likely to be killed by a police officer than whites individuals (Economist, 2020). Alicia Garza, the prominent American civil rights activist, author and co-founder of the Black Lives Matter movement has stated, “The fight is not just being able to keep breathing. The fight is actually to be able to walk down the street with your head held high—and feel like I belong here, or I deserve to be here, or I just have [a] right to have a level of dignity (Denzel Smith, 2015).” To be white is to roam freely, but to be a person of color is to walk a shackled path.

As noted elsewhere (Hackett, 2020a) being white has afforded the first author many privileges that he is often not even aware of. For example, Christian Cooper was a bird watcher of color who was subject to a verbal racial attack whilst he was watching birds in Central Park, New York, last year (see, Axelson, 2020; Mock, 2020). The first author has also watched birds in locations across the world and “occasionally has felt conspicuous when using his binoculars and even nervous about how he may be perceived by others.” He provided the example of many years ago bird watching in communist Eastern Europe as being a time when he was wary. When he has watched birds in Central Park, the same location as Christian Cooper, he felt a little apprehensive due to the fact that he constituted an unusual sight. However, he noted that: “In Central Park (I have), never been afraid of being labeled a White man; a person who is “not of color.” In this and other situations my color is transparent, my color is seen (white) and this is perceived as being of no threat. It is as if I have no color. I am just a birdwatcher.

However, Christian Cooper was labeled an African American male by an irate dog owner and even though this is a politically correct epithet it was used to describe Cooper to the police it labeled him with a term of difference, a term of racial abuse.

The preceding paragraphs constitute an extremely selective and brief reflection upon racism in contemporary US society. Obviously, the examples of racism are broader, more varied and wider ranging than those we have given. Above, we have, on a few occasions, used the term racialized as it avoids the ascription of intent to an activity or event that has negative outcomes for people of color or of a minority race. This is an important point as using the term racialized avoids having to determine whether an action was committed with an intended negative racial outcome. A racialized event or action is simply one that differentiates on a color, racial or minority basis and typically has a negative outcome for members of these groups regardless of purpose or objective.

## Identifying Racist and Racialised Actions and evets

In recent decades numerous scholars have attempted to identify and to define racism and racialized activity. Some of these researchers have employed an intersectional lens to explore the expansive and multifarious nature of institutional racism (Sabik, 2021). Unfortunately, the scope of this paper does not allow sufficient space for us to fully reflect upon key contemporary concepts presented within Critical Race Theory (e.g., Delgado and Stephancic, 2017) and intersectionality scholarship or their direct correlation to our earlier stated cases of racism and racialization. However, we acknowledge this often seminal and extremely important work by black academics such as Crenshaw et al. (1996), Jones (2014, 2018); Ford (2019), and Jones (2000).

## Declarative Mapping Sentence

With this in mind, the aim of this brief paper is to present a framework for describing racist or racialized expressions using the declarative mapping sentence method: an approach which embodies the complexity of racism and offers a tool for designing research into different forms of racism and racialized events. The DMS we present came out of extensive literature search and discussions between the two authors and forms a cumulative synthesis of much other work we have undertaken that considers racist and racialized expressions.

**Figure 1:**
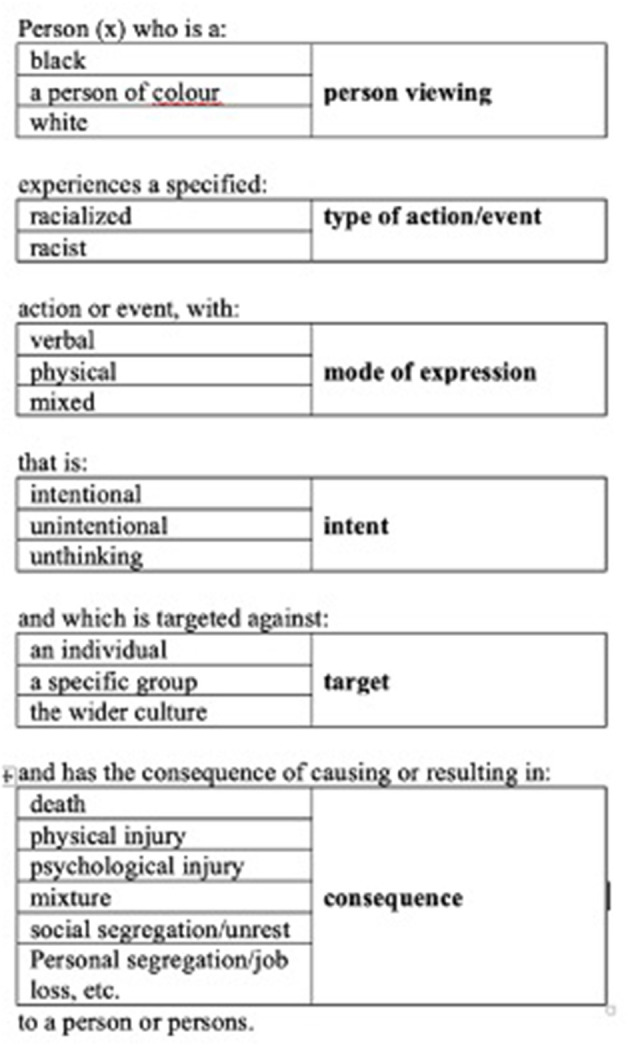
Declarative mapping sentence for understanding acts of racism (adapted from Hackett and Schwarzenbach, 2020).

The declarative mapping sentence is a core methodology of Facet Theory. Facet Theory was developed over eight decades ago as an attempt to combine theory with the data collected whilst providing apt analysis (Guttman, 1944, 1954, 1957; Canter, 1985; Hackett, 2021). The declarative mapping sentence method has been employed in several research contexts (e.g., Hackett, 2020a,b, 2021) as a framework within which qualitative research may be designed and conducted. It is both an inductive and deductive approach as it provides a flexible and responsive framework for research whilst being constantly updated based upon the experiences of the researcher. Below we propose a declarative mapping sentence (DMS) that is an adaptation of earlier research (Hackett and Schwarzenbach, 2020) which attempts to provide a framework for understanding and defining racist and racialized acts (Figure 1). This framework may be used to design and interpret research in this area. Employing the DMS in the development of a research project enables the inclusion of the important aspects of such acts within the research. There are six facets in this declarative mapping sentence for understanding acts of racism (written in the right column). These are: mode of person viewing, type of action/event, mode of expression, intent, target, consequence. To the left of each of these six facets are written their respective elements.

The DMS states that racist acts are committed by people, either individually or in groups. The sentence specifies that such an act may be thought of in terms of the type of action committed which are specified as being verbal, physical, or acts that are a mixture of these. The DMS then denotes that it is important to consider the intention behind such an action, which may be intentional, unintentional, or unthinking. Who is the action targeted against is of importance and in the DMS the targets may be individual, specific groups, or wider culture groups. The consequence of the action is also important to keep in mind when designing and interpreting research: we suggest the elements of death, physical injury, psychological injury, a mixture of these, and social segregation/unrest. It is also noted that the person who is viewing and interpreting the action is an important component of the research in terms of whether they are Black or not Black. It is our contention that it is important to consider these aspects of the situation when designing or interpreting a piece of qualitative research into racism.

The DMS may be used as a design template and a coding frame. When using this as a designing tool observations and questions are developed based upon elements from all of the facets combined in a manner suggested in the DMS. This allows the researcher to be assured that all of the pertinent aspects of the research area are included in their inquiries. When used as a coding frame during thematic content analysis, the elements of the mapping sentence are used in a researcher's first attempt at coding a specific data set. Schreier (2012) suggests that coding should be undertaken using three attempts of ever greater finesse. When using the declarative mapping sentence to code data the elements are observed for on the first reading and then modified on the subsequent two rounds of coding and as the codes become more concrete and contextualized, so the elements of the mapping sentence are deleted, added to and amended.

## Author's Note

The authors of this chapter acknowledge that they are white, middle class individuals and that this background inevitably impacts their appreciation of racism and influences how they write about racist issues.

## Author Contributions

All authors listed have made a substantial, direct, and intellectual contribution to the work and approved it for publication.

## Conflict of Interest

The authors declare that the research was conducted in the absence of any commercial or financial relationships that could be construed as a potential conflict of interest.

## Publisher's Note

All claims expressed in this article are solely those of the authors and do not necessarily represent those of their affiliated organizations, or those of the publisher, the editors and the reviewers. Any product that may be evaluated in this article, or claim that may be made by its manufacturer, is not guaranteed or endorsed by the publisher.
